# Characteristics and clinical features of cauda equina syndrome: insights from a study on 256 patients

**DOI:** 10.1051/sicotj/2023019

**Published:** 2023-07-19

**Authors:** Junaid Zeb, Jehan Zaib, Arshad Khan, Mehreen Farid, Seemab Ambreen, Syed Hussaini Shah

**Affiliations:** 1 Registrar Trauma and Orthopaedics, Russells Hall Hospital, The Dudley Group NHS Foundation Trust Pensnett Rd Dudley DY1 2HQ United Kingdom; 2 Associate Specialist, Trauma and Orthopaedics, Russells Hall Hospital, The Dudley Group NHS Foundation Trust Pensnett Rd Dudley DY1 2HQ United Kingdom; 3 SHO Trauma and Orthopaedics, Russells Hall Hospital, The Dudley Group NHS Foundation Trust Pensnett Rd Dudley DY1 2HQ United Kingdom; 4 Clinical Attachment Trauma and Orthopedics, Russells Hall Hospital, The Dudley Group NHS Foundation Trust Pensnett Rd Dudley DY1 2HQ United Kingdom

**Keywords:** Cauda equina syndrome, Clinical presentation, Etiological factors, Neurological deficit

## Abstract

*Objective*: To determine the frequency, clinical presentation, and etiological factors of cauda equina syndrome (CES). *Materials and method*: This retrospective study was done on 256 participants, and aimed to analyze the frequency and patterns of clinical presentation in suspected cases of CES. The inclusion criteria included participants aged 18 or older with medical records available for review and having red-flagged symptoms for CES. The study collected information on various factors such as age, gender, confirmation of CES on MRI, neurological deficits, etiological factors, duration of symptoms, and more. The data collected was analyzed using descriptive statistics and logistic regression to identify significant variables between MRI-proven CES and suspected CES. *Results*: The mean age was 58.05 ± 19.26 years, with 151 females (58.98%) and 105 males (41.02%). The majority (50.78%) had a neurological deficit, while other symptoms included difficulty initiating micturition or impaired sensation of urinary flow (17.58%), loss of sensation of rectal fullness (3.12%), urinary or faecal incontinence (35.16%), bilateral sciatica (21.88%), neurological symptoms in the lower limbs (25.00%), anaesthesia or any leg weakness (24.22%), and bilateral sciatica as the predominant symptom (21.88%). Symptoms were chronic in 47.27% and acute in 21.88%. The odds of MRI-proven CES increase by 3% per year of age. Neurological deficit was strongly associated with MRI-proven CES (OR = 14.97), while loss of sensation of rectal fullness increased the odds by 10-fold (OR = 10.62). *Conclusion*: CES can present with various symptoms, including the bilateral neurological deficit, urinary and faecal incontinence, and bilateral sciatica, with age, severe bilateral neurological deficit, and loss of sensation of rectal fullness being associated with MRI-proven CES. Early diagnosis and treatment are crucial for better outcomes.

## Introduction

Cauda equina syndrome (CES) is a rare but serious neurological condition that occurs when the bundle of nerve roots at the bottom of the spinal cord, known as the cauda equina, is compressed or damaged [1]. CES is typically marked by several key symptoms, commonly referred to as “red flags”, including severe lower back pain, bilateral or absent sciatica, sensory disturbances in the saddle and/or genital area, and dysfunction of the bladder, bowel, and sexual organs [2].

Diagnosis of CES is typically made through a combination of physical exams, imaging studies, and nerve function tests. A systemic review showed that currently, there is no consensus on clinical diagnostic criteria for CES [3]. The lack of clear diagnostic criteria for CES and common symptoms in the population leads to low CES diagnosis rates on MRI when suspected, making it challenging to accurately diagnose and potentially resulting in delayed treatment and negative outcomes for patients [4, 5].

Earlier investigations have documented the occurrence frequencies of CES, which have varied from 0.3 to 7.0 cases per 100,000 individuals annually [1]. A systematic review including four studies aimed to determine if elements from the history or physical examination are associated with CES as established by MRI. Their results showed that the average prevalence of CES, diagnosed by MRI, ranged from 14% to 48% among patients. And no signs and symptoms were diagnostics for CES diagnosis [6].

The literature commonly reports back pain, bowel incontinence, bilateral sciatica, bladder incontinence, bladder retention, decreased urinary sensation, and frequent urination as symptoms of CES [7–9]. Additionally, CES is frequently associated with saddle numbness, reduced anal tone, decreased ankle reflex, and loss of power [10].

CES is a rare but potentially devastating condition that can result in permanent neurological damage if not promptly diagnosed and treated. Understanding the frequency, clinical presentation and underlying causes of this syndrome is crucial for healthcare providers to identify and manage cases effectively. Therefore, the rationale for this retrospective study is to provide insights into the epidemiology, clinical features, and etiological factors of CES, which could help improve its diagnosis and management in clinical practice. By examining a large number of cases retrospectively, this study can provide a comprehensive understanding of the characteristics and outcomes of CES and ultimately contribute to better clinical decision-making and patient care.

The purpose of this study was to ascertain the frequency, clinical presentation, and etiological factors of CES.

## Methodology

This retrospective observational study was conducted on 256 participants at the Department of Orthopaedics, Russell’s Hall Hospital, United Kingdom, using available electronic patient records from 1st January 2022 to 30th June 2022. Patient confidentiality was maintained throughout the study, and the data collected was used for research purposes only. The study protocol was registered with the hospital audit committee via AMaT before the data collection was started. Informed consent was not required as this was a retrospective study, and the data were anonymized.

The inclusion criteria were as follows: participants aged 18 years or older, either gender, medical records available for review, and suspected CES (having any of the red-flagged symptoms, such as severe lower back pain, bilateral or absent sciatica, sensory disturbances in the saddle and/or genital area, and dysfunction of the bladder, bowel, and sexual organs). The following information was collected: age (years), gender, confirmation of CES on MRI, neurological deficit, micturition problems, loss of sensation of rectal fullness, urinary or faecal incontinence, bilateral sciatica, neurological symptoms in the lower limbs, past history and severity of symptoms, etiological factors for CES (previous medical history (PMH), history of cancer or trauma, previous education about CES), duration of symptoms, volume on bladder scan, and need for catheterization.

The data collected were entered into Microsoft Excel sheet 2016 and imported into R programming 4.1.2 for analysis. Descriptive statistics, such as means, standard deviations, and percentages, were used to summarize the data. The chi-square test was applied to compare symptoms and etiological factors among MRI-proven CES versus suspected CES. The significant variables among symptoms and etiological factors among MRI-proven CES versus suspected CES were run by logistic regression using CES (MRI-proven versus suspected) as the dependent variable to calculate odds ratios with 95% CI. The significance level was set at 0.05.

## Results

The mean age of the participants was 58.05 ± 19.26 years. Table 1 shows data pertaining to 256 individuals and presents information on their gender and age group. In terms of gender, there were 151 females (58.98%) and 105 males (41.02%) in the sample. Regarding age group, the largest proportion of individuals fell within the 51–70 age range (31.25%, *n* = 80), followed by those aged 70 and above (33.59%, *n* = 86), 31–50 age range (27.73%, *n* = 71), and the smallest proportion within the 16–30 age range (7.42%, *n* = 19).

**Table 1:** Gender and age distribution of the study.

Variable	Characteristic	*N* = 256
Gender	Female	151 (58.98)
	Male	105 (41.02)
Age group	16–30	19 (7.42)
	31–50	71 (27.73)
	51–70	80 (31.25)
	70 and above	86 (33.59)

The study included 256 participants, of whom 50.78% had a severe or progressive bilateral neurological deficit, 17.58% had difficulty initiating micturition or impaired sensation of urinary flow, 3.12% had a loss of sensation of rectal fullness, 35.16% had urinary or faecal incontinence, 21.88% had bilateral sciatica, 25.00% had neurological symptoms in the lower limbs, 24.22% had anaesthesia or any leg weakness, and 21.88% had bilateral sciatica as the predominant symptom. The symptoms were acute in 21.88% of the participants, chronic in 47.27%, and absent in 51.95%. The rest of the overall results are given in Table 2. The frequency of severe or progressive bilateral neurological deficit was significantly higher in patients with MRI-proven CES (19.5%, *n* = 25) compared to those with suspected CES (44.98%) and the overall population (50.78%) (*p* < 0.001). The frequency of difficulty initiating micturition or impaired sensation of urinary flow, bilateral sciatica, predominant bilateral sciatica symptom, change in the severity of symptoms, the time point for change in the severity of symptoms, investigation in past for the same symptoms, did symptoms start with the back, and radicular leg pain did not differ significantly between the two groups. However, the frequency of loss of sensation of rectal fullness was significantly higher in MRI-proven CES (11.11%) compared to suspected CES (2.18%) (*p* = 0.05). Similarly, the frequency of urinary or faecal incontinence was higher in MRI-proven CES (44.44%) compared to suspected CES (34.06%), although the difference was not significant (*p* = 0.392). The frequency of neurological symptoms in the lower limbs and anaesthesia or any leg weakness did not differ significantly between the two groups. In addition, there was no significant difference in the time point for change in the severity of symptoms, investigation in past for same symptoms, did symptoms start with the back, and radicular leg pain between the two groups. The frequency of neurological symptoms was higher in MRI-proven CES (88.89%) compared to suspected CES (79.04%), although the difference was not significant (*p* = 0.338) (Table 2).

**Table 2:** Comparison of clinical characteristics between suspected and MRI-proven CES patients.

Variable	Characteristic	Overall, *N* = 256	Suspected CES, *N* = 229	MRI-proven CES, *N* = 27	*P*-value
Severe or progressive bilateral neurological deficit	Present	128 (50)	103 (44.98)	25 (19.5)	<0.001
Difficulty initiating micturition or impaired sensation of urinary flow	Present	45 (17.58)	42 (18.34)	3 (11.11)	0.505
Loss of sensation of rectal fullness	Present	8 (3.12)	5 (2.18)	3 (11.11)	0.05
Urinary or faecal incontinence	Present	90 (35.16)	78 (34.06)	12 (44.44)	0.392
Bilateral sciatica	Present	56 (21.88)	54 (23.58)	8 (29.63)	0.648
Neurological symptoms in the lower limbs	Present	64 (25.00)	169 (73.80)	23 (85.19)	0.29
Anaesthesia or any leg weakness	Present	62 (24.22)	173 (75.55)	21 (77.78)	0.985
Was B/L sciatica the predominant symptom	Present	56 (21.88)	48 (20.96)	8 (29.63)	0.433
Timeline of the symptoms (i.e. acute/chronic)	Acute	56 (21.88)	108 (47.16)	13 (48.15)	0.887
	Chronic	121 (47.27)	119 (51.97)	14 (51.85)	
	No	133 (51.95)	2 (0.87)	0 (0.00)	
Was this the first episode of symptoms or have they had it before	Before	2 (0.78)	111 (48.47)	14 (51.85)	0.898
	First	125 (48.83)	117 (51.09)	13 (48.15)	
	No	130 (50.78)	1 (0.44)	0 (0.00)	
Change in severity of symptom	present	151 (58.98)	91 (39.74)	14 (51.85)	0.316
Time point for change in severity of symptom	NA	149 (58.20)	135 (58.95)	14 (51.85)	0.675
	Since <1 week	32 (12.50)	28 (12.23)	4 (14.81)	
	Since 1–3 weeks	55 (21.48)	47 (20.52)	8 (29.63)	
	Since 1 month	12 (4.69)	12 (5.24)	0 (0.00)	
	Since 2–3 months	6 (2.34)	5 (2.18)	1 (3.70)	
	Since 6 months	2 (0.78)	2 (0.87)	0 (0.00)	
Investigation in past for same symptoms	Present	102 (39.84)	91 (39.74)	11 (40.74)	>0.999
Did the symptoms start with the back	Present	201 (78.52)	182 (79.48)	19 (70.37)	0.4
Radicular leg pain	Present	136 (53.12)	120 (52.40)	16 (59.26)	0.637
Neurological symptoms	Present	205 (80.08)	181 (79.04)	24 (88.89)	0.338

**Fig. 1:**
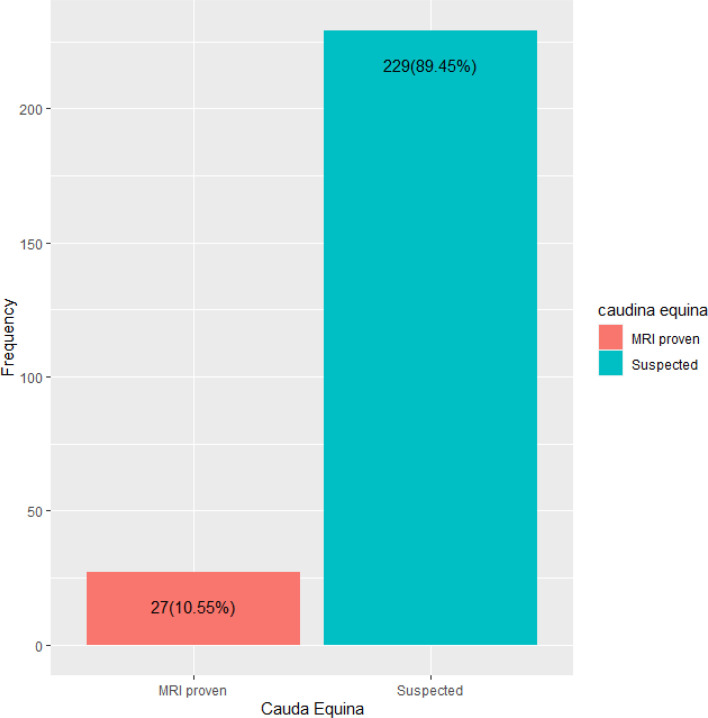
Frequency of cauda equina.

Among all 10.55% (27) had MRI-proven CES while 89.45% (229) had suspected CES (Figure 1). Among the 256 participants, 48.05% had a PMH of cancer diagnosis or previous CES, 21.48% had a history of trauma, 10.55% had a PMH of previous CES, and only 1.56% was previously given information about CES. In addition, 61.72% of patients received instructions about changing the consequences of delayed care. The majority of participants (42.19%) had symptoms lasting between 72 h and 2 weeks, while 36.33% had symptoms lasting more than 2 weeks, and 19.53% had symptoms lasting less than 72 h. Among those for whom it was available, 6.64% had a volume of 400 mL and above, 2.73% had a volume of 200–400 mL, 5.86% had a volume of 100–200 mL, and 19.53% had a volume of less than 100 mL. Finally, 26.17% of participants required catheterization. There was no significant difference in the history of cancer diagnosis or previous medical history (*p* = 0.549) or history of trauma in the past (*p* = 0.882) between patients with MRI-proven CES and those with suspected CES. However, patients with MRI-proven CES had a higher likelihood of a previous history of CES than those with suspected CES (*p* = 0.016). Patients with MRI-proven CES were significantly more likely to receive instructions about the changing consequences of delayed care (*p* = 0.004) and had a significantly higher post-micturition bladder volume (*p* < 0.001) with a higher likelihood of a bladder volume of 400 mL or above (*p* = 0.003). Non-progressive symptoms lasting 72 h–2 weeks had a higher likelihood of MRI-proven CES (*p* = 0.137). No significant difference existed in the need for catheterization (*p* = 0.507) between patients with MRI-proven CES and those with suspected CES (Table 3).

**Table 3:** Overall frequency and comparison of etiological factors for suspected and MRI-proven CES.

Variable	Characteristic	Overall, *N* = 256	Suspected CES, *N* = 229	MRI-proven, *N* = 27	*P*-value
PMH of cancer diagnosis, or previous CES	Yes	123 (48.05)	112 (48.91)	11 (40.74)	0.549
Hx of trauma in past	Yes	55 (21.48)	50 (21.83)	5 (18.52)	0.882
Is there a PMH of previous CES	Yes	27 (10.55)	20 (8.73)	7 (25.93)	0.016
Patient was given any information about cauda equina syndrome previously	Yes	4 (1.56)	3 (1.31)	1 (3.70)	0.898
Instruction about changing consequences in delayed care	Yes	158 (61.72)	134 (58.52)	24 (88.89)	0.004
Duration of symptoms	<72 h progressive symptom	55 (21.48)	52 (22.71)	3 (11.11)	0.137
	>2/52	93 (36.33)	85 (37.12)	8 (29.63)	
	72 h–2 weeks/non-progressive symptom	108 (42.19)	92 (40.17)	16 (59.26)	
Post-micturition bladder volume (mL)	<100	50 (19.53)	50 (21.83)	0 (0.00)	<0.001
	100–200	15 (5.86)	15 (6.55)	0 (0.00)	
	200–400	7 (2.73)	3 (1.31)	4 (14.81)	
	400 and above	17 (6.64)	8 (3.49)	9 (33.33)	
	Not available	167 (65.23)	153 (66.81)	14 (51.85)	
Need of catheterization	Yes	67 (26.17)	58 (25.33)	9 (33.33)	0.507

The multivariable analysis adjusts for the effects of other variables on the outcome (CES type). The OR for age in the multivariable analysis is 1.03, indicating that, after adjusting for other variables, the odds of MRI-proven CES increase by 3% for each one-year increase in age. Gender is not significantly associated with MRI-proven CES in either univariable or multivariable analysis. The OR for severe or progressive bilateral neurological deficit in the multivariable analysis is 14.97, indicating that, after adjusting for other variables; patients with this condition are almost 15 times more likely to have MRI-proven CES compared to those without this condition. The OR for loss of sensation of rectal fullness in the multivariable analysis is 10.62, indicating that, after adjusting for other variables, patients with this condition are over 10 times more likely to have MRI-proven as compared to suspected (Table 4).

**Table 4:** Multivariable analysis of the factors associated with MRI-proven CES.

Variable	Characteristics	Cauda equina		OR (univariable)	OR (multivariable)
		Suspected	MRI-proven		
Age	Mean (*SD*)	57.4 (19.5)	63.9 (15.8)	1.02 (1.00–1.04, *p* = 0.101)	1.03 (1.00–1.06, *p* = 0.066)
Gender	Female	138 (91.4)	13 (8.6)	–	–
	Male	91 (86.7)	14 (13.3)	1.63 (0.73–3.68, *p* = 0.229)	1.77 (0.73–4.40, *p* = 0.208)
Severe or progressive bilateral neurological deficit	Absent	126 (98.4)	2 (1.6)	–	–
	Present	103 (80.5)	25 (19.5)	15.29 (4.42–96.44, *p* < 0.001)	14.97 (4.15–96.79, *p* < 0.001)
Loss of sensation of rectal fullness	Absent	224 (90.3)	24 (9.7)	–	–
	Present	5 (62.5)	3 (37.5)	5.60 (1.10–24.30, *p* = 0.024)	10.62 (1.49–92.15, *p* = 0.020)
Is there a PMH of CES	No	209 (91.3)	20 (8.7)	–	–
	Yes	20 (74.1)	7 (25.9)	3.66 (1.31–9.43, *p* = 0.009)	3.03 (0.98–8.96, *p* = 0.047)
Written information given about CES previously	No	226 (89.7)	26 (10.3)	–	–
	Yes	3 (75.0)	1 (25.0)	2.90 (0.14–23.58, *p* = 0.364)	4.77 (0.21–57.72, *p* = 0.228)

## Discussion

The study analyzed 256 individuals with varying symptoms of CES. Among them, 10.55% had MRI-proven CES, while 89.45% had suspected CES. Patients with MRI-proven CES had a higher likelihood of previous history of CES, receiving instructions about the changing consequences of delayed care, and had a significantly higher post-micturition bladder volume. Our study has revealed a significant relationship between a patient’s previous history of CES and the presence of confirmed CES on MRI. One possible explanation for this association could be that patients with a history of CES have experienced a prolonged duration of the condition, leading them to seek medical attention and undergo MRI as part of the diagnostic process. Healthcare providers should evaluate patients’ medical histories carefully and consider the possibility of underlying conditions that may be contributing to their current symptoms.

Females (58.98%) were more affected by CES than males (41.02%). This finding is consistent with a previous study conducted on the Brazilian population, which reported a gender distribution of 13 female patients (59%) and nine male patients (41%) [11]. The mean age of the participants was 58.05 ± 19.26 years. CES is commonly associated with degenerative spinal changes that are more likely to occur in the elderly population. A previous study conducted in Brazil reported a significantly lower mean age of 44.16 ± 12.83 years among the patients with CES, ranging from 22 to 64 years [11]. In contrary, male predominance for CES was reported in other studies too [12, 13]. Many factors play a role in delayed presentations among CES patients. The lack of knowledge among healthcare professionals about the classic signs and symptoms of CES can result in delayed diagnosis [14]. This problem is further compounded by the inadequate diagnostic resources in primary healthcare services and socioeconomic factors that limit access to healthcare [15]. Overcrowding and long wait times at public healthcare services exacerbate the situation, highlighting the need for increased investment in healthcare resources to provide timely and effective care to patients with CES [16].

The severe or progressive bilateral neurological deficit, urinary or faecal incontinence, bilateral sciatica, and neurological symptoms in the lower limbs were the most common clinical features observed among the study participants. Literature reports that a considerable number of patients with CES may experience long-term neurological sequelae. These sequelae can range from permanent motor and sensory deficits to neurogenic bladder, which was observed in 64% of patients in one study [17]. Additionally, 36% of patients in another study showed persistence of the initial deficit [18].

The multivariate analysis shows that age was found to be significantly associated with MRI-proven CES, with odds increasing by 3% for each one-year increase. Severe or progressive bilateral neurological deficit and loss of sensation of rectal fullness were strongly associated with MRI-proven CES, with patients with these conditions almost 15 and over 10 times more likely to have MRI-proven CES, respectively, compared to those without or with suspected CES. Literature shows that MRI should always be considered for diagnosing CES [19].

## Conclusion

In conclusion, our study provides important insights into the clinical features of CES, which can aid healthcare providers in the timely diagnosis and management of the condition, potentially mitigating its long-term neurological sequelae. Future studies can build on our findings to develop more effective diagnostic and management strategies for CES.
